# Editorial: Anxiety in autistic people: moving the needle

**DOI:** 10.3389/fpsyt.2026.1947934

**Published:** 2026-09-04

**Authors:** Alana J. McVey, Carla Wall

**Affiliations:** 1 Center for Behavioral Medicine, Brookfield, WI, United States; 2 Department of Psychology, Marquette University, Milwaukee, WI, United States; 3 Department of Psychiatry, Children’s Hospital of Richmond at Virginia Commonwealth University, Richmond, VA, United States

**Keywords:** anxiety, autism, measurement, neurodiversity, treatment

## Introduction

This Research Topic is a collection of recent developments in the study of anxiety in Autistic people. Nearly 10 years ago, another collection summarized mechanisms, measurement, and treatment in this area (1)—we are pleased to present several notable advances. Studies here examined factors across additional diagnoses (depression, ADHD, psychotic-like experiences) to determine common and distinct mechanisms (2–5). Advances in psychological research more broadly—including multi-informant assessment (6) and implementation of evidence-based practices in school settings (7)—are reflected here (8, 9). Brook’s (10) novel application of the ASCENT model to exposure therapy is reflective of a broader mind shift in the autism field from a medicalized to a social disability view of autism (11) and provides clinicians with a framework for Autism-affirming treatment. Together, these papers reflect meaningful progress in the study of anxiety in Autistic people, grounded in an increasingly affirming approach to the subject matter.

## Mechanisms and moderators

Hunsche et al. used parallel process latent growth curve models to examine joint trajectories of caregiver-reported anxiety and depression symptoms in a longitudinal sample of Autistic youth (ages 7 to 16 years) with a range of communication abilities. Anxiety symptoms did not change significantly on average, while depression symptoms increased over time—although there was considerable variability across individuals. Notably, youth who changed in one domain tended to change in the other. More autism characteristics and greater emotional reactivity predicted higher levels of initial anxiety and depression symptoms, but not their trajectories. A key takeaway is the importance of jointly monitoring both anxiety and depression in this developmental period.

To investigate the role of cognitive flexibility in anxiety symptoms, Mahmud et al. used multiple linear regressions to examine associations between anxiety and both lab-based task switching performance and real-world flexibility in a sample of Autistic children (ages 8–17 years). They found that lower levels of real-world flexibility predicted anxiety independent of autism characteristics, while lab-based task switching performance did not.

In their systematic review, Rowe et al. investigated binary links among anxiety, autism, and psychotic-like experiences and found that both anxiety and autism independently predicted psychotic-like experiences. They concluded that Autistic people with co-occurring anxiety disorders may experience a greater risk for psychotic-like experiences—meriting further empirical study.

In a sample of 3-to-5-year-old Autistic children with anxiety and/or ADHD, Waters et al. examined the role of executive functions. Using an additive main effect general linear model and mediation analyses, they found difficulties with attentional shifting were specifically linked with anxiety, while difficulties inhibiting responses were specifically linked with ADHD. Additionally, attentional shifting mediated the relationship between anxiety and several specific autism characteristics (repetitive behaviors, sameness behaviors, sensory hypersensitivity, and overall autism features) while inhibitory control mediated the relationship between ADHD and irritability and self-injury.

## Measurement

Coffman et al. used repeated measures ANOVAs to test for discrepancies in caregiver- and adolescent self-report of social anxiety on the Anxiety Disorders Interview Schedule for DSM-5 (ADIS-5) (12) in a sample of Autistic adolescents with social anxiety, allistic adolescents with social anxiety, and a comparison group. Results showed that caregivers consistently reported greater interference and severity than adolescents did—this pattern was most pronounced in the Autistic group. Additionally, polynomial regressions found that better adaptive functioning was associated with greater agreement in the Autistic group but greater disagreement in the social anxiety group. Clinician judgment appeared to buffer some of the discrepancies, which points to the importance of integrated multi-informant assessment and the role of trained clinicians in integrating discrepant reports.

Two studies used anxiety tools developed specifically for Autistic populations. In a community sample of Autistic children and adolescents (8–17 years of age), Mahmud et al. collected self- and parent-reported anxiety using the Anxiety Scale for Children with Autism Spectrum Disorder (13). Self-reported anxiety symptoms were significantly higher than parent-reported symptoms—a directional discrepancy that differs from prior findings with this measure in clinical samples. Reaven et al. extended the evidence base for the ADIS with Autism Spectrum Addendum (ADIS/ASA) (14) by demonstrating its feasibility in a school-based setting and its utility in capturing anxiety presentations that other rating scales would have missed. They also demonstrated its responsiveness to intervention-related change and strong interrater reliability in a real-world setting, supporting its use as both a diagnostic and outcome measure.

## Treatment

Two papers focused on the treatment of anxiety in Autistic people. Drawing on lived and clinical expertise, Brook proposed the application of a model originally developed for Dialectical Behavior Therapy (15) to guide autism-affirming adjustments to exposure therapy in the treatment of anxiety disorders. Grounded in an affirming view of autism and centered on a client’s goals, the ASCENT (Autonomous and affirming goal setting; Sensory, stimming, structure, special interests; Communication; Executive functioning; Neurohumility; Trauma-informed) model provides clinicians with guidance to parse anxiety symptoms from autism characteristics and accommodate the latter. By using this model, therapists may be more likely to deliver exposure therapy for anxiety in a manner that is affirming and supportive of an Autistic identity.

In a sample of students aged 7–14 years who were or suspected to be Autistic, Reaven et al. found that Facing Your Fears in Schools (FYF-S) (16) reduced DSM-5 anxiety disorder symptoms with less clear effects on distinct anxiety presentations. This study provided further evidence for the effectiveness of FYF-S in real-world school settings using a more rigorous outcome measure (ADIS/ASA) (14).

## Future directions

Gaps remain in the field and are reflected in our Research Topic. First, Brook was the sole paper in our Research Topic to include a focus on Autistic adults. This is a notable gap, as meta-analytic evidence demonstrates that the lifetime prevalence of anxiety disorders in Autistic adults is approximately 42% (17) and there are critical barriers to accessing such services (18). Second, studies here did not include participants with co-occurring intellectual disability, genetic syndromes, and/or those who are nonspeaking—populations that together represent a substantial proportion of Autistic people (19–21) and among whom anxiety symptoms may be similarly prevalent (22). This remains a key area of future research. Third, papers did not report community collaboration or work led by Autistic researchers. Community-partnered and Autistic-led research has been increasingly recognized as essential to producing research that is relevant and meaningful to Autistic people (23). Lastly, none of the studies in our Research Topic directly tested factors that confer risk for or resilience against the development of anxiety disorders—representing an important direction for future work to improve outcomes for this population.

## Conclusion

Taken together, this Research Topic provides a window into priorities for research on anxiety in Autistic people. More research is needed to examine key mechanisms longitudinally (including mediators and moderators) and to advance and validate measurement tools for this population. Additionally, outcome studies of Autism-affirming treatments across age groups and ability levels will expand access to effective care for this population. Lastly, work that is led or co-led by Autistic researchers is paramount to ensuring the priorities of the community are centered.
